# Endovascular Thrombectomy for Large Ischemic Strokes with ASPECTS 0–2: a Meta-analysis of Randomized Controlled Trials

**DOI:** 10.1007/s00062-024-01414-2

**Published:** 2024-04-30

**Authors:** Laurens Winkelmeier, Máté Maros, Fabian Flottmann, Christian Heitkamp, Gerhard Schön, Götz Thomalla, Jens Fiehler, Uta Hanning

**Affiliations:** 1 Department of Neuroradiology, University Medical Center Hamburg-Eppendorf, Hamburg, Germany; 2 Department of Biomedical Informatics, Medical Faculty Mannheim, Heidelberg University, Mannheim, Germany; 3 Institute for Medical Biometry and Epidemiology, University Medical Center Hamburg-Eppendorf, Hamburg, Germany; 4 Department of Neurology, University Medical Center Hamburg-Eppendorf, Hamburg, Germany

**Keywords:** Cerebral infarction, Infarction, Ischemic stroke, Stroke, Thrombectomy

## Abstract

**Purpose:**

Randomized controlled trials (RCTs) demonstrated a treatment effect of endovascular thrombectomy in acute ischemic stroke with large infarct, commonly defined as an Alberta Stroke Program Early CT Score (ASPECTS) of 3–5. However, data on endovascular thrombectomy in patients with very low ASPECTS of 0–2 remain scarce.

**Methods:**

We conducted a systematic review and meta-analysis of RCTs comparing endovascular thrombectomy versus medical treatment alone in acute ischemic anterior circulation stroke with very large infarct, defined as ASPECTS of 0–2. The primary outcome was the shift toward better functional outcomes on the 90-day modified Rankin Scale (mRS). Random effects meta-analysis was performed using the generic inverse variance method.

**Results:**

Literature research identified four RCTs which evaluated the treatment effect of endovascular thrombectomy for large infarcts and provided a subgroup analysis of the mRS shift in patients with ASPECTS of 0–2. The pooled analysis showed a significant shift toward better 90-day mRS scores in favor of endovascular thrombectomy (pooled odds ratio, 1.62, 95% confidence interval, 1.29–2.04, *P* < 0.001).

**Conclusion:**

This meta-analysis suggests a treatment effect of endovascular thrombectomy in specific patients with very low ASPECTS of 0–2, challenging the use of ASPECTS for treatment selection in acute ischemic stroke due to large vessel occlusion. An individual patient meta-analysis of RCTs would strengthen evidence in the treatment of patients with ASPECTS of 0–2.

**Graphic abstract:**

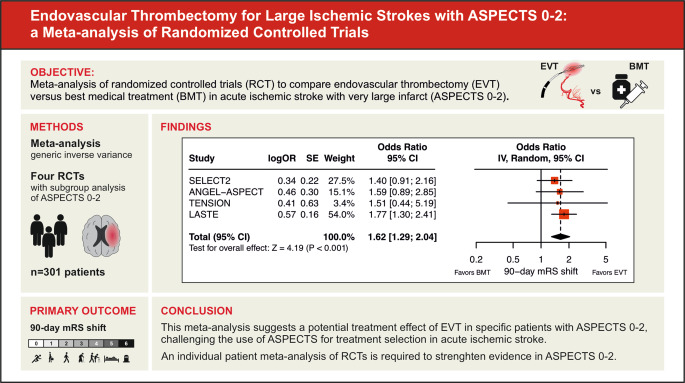

## Introduction

Multiple randomized controlled trials (RCTs) showed efficacy of endovascular thrombectomy (EVT) over medical management alone in patients with acute ischemic stroke due to anterior circulation large vessel occlusion and already established large infarct [1–5]. These trials largely enrolled patients with an Alberta Stroke Program Early CT Score (ASPECTS) of 3–5, for which a treatment effect of EVT can now be assumed with high certainty.

In contrast, uncertainties remain about the benefit of EVT in patients with very low ASPECTS of 0–2. Recently, the LASTE trial provided strong evidence that EVT is efficacious and safe in specific patients with ASPECTS of 0–2 [5]. Other RCTs with differing inclusion criteria reported a tendency to improved functional outcomes in patients with very low ASPECTS who received EVT, but were underpowered to provide precise estimates for this highly affected subgroup.

We performed a meta-analysis to investigate the treatment effect of EVT in patients with ASPECTS of 0–2 across RCTs.

## Materials and Methods

### Study Design

**Fig. 1:**
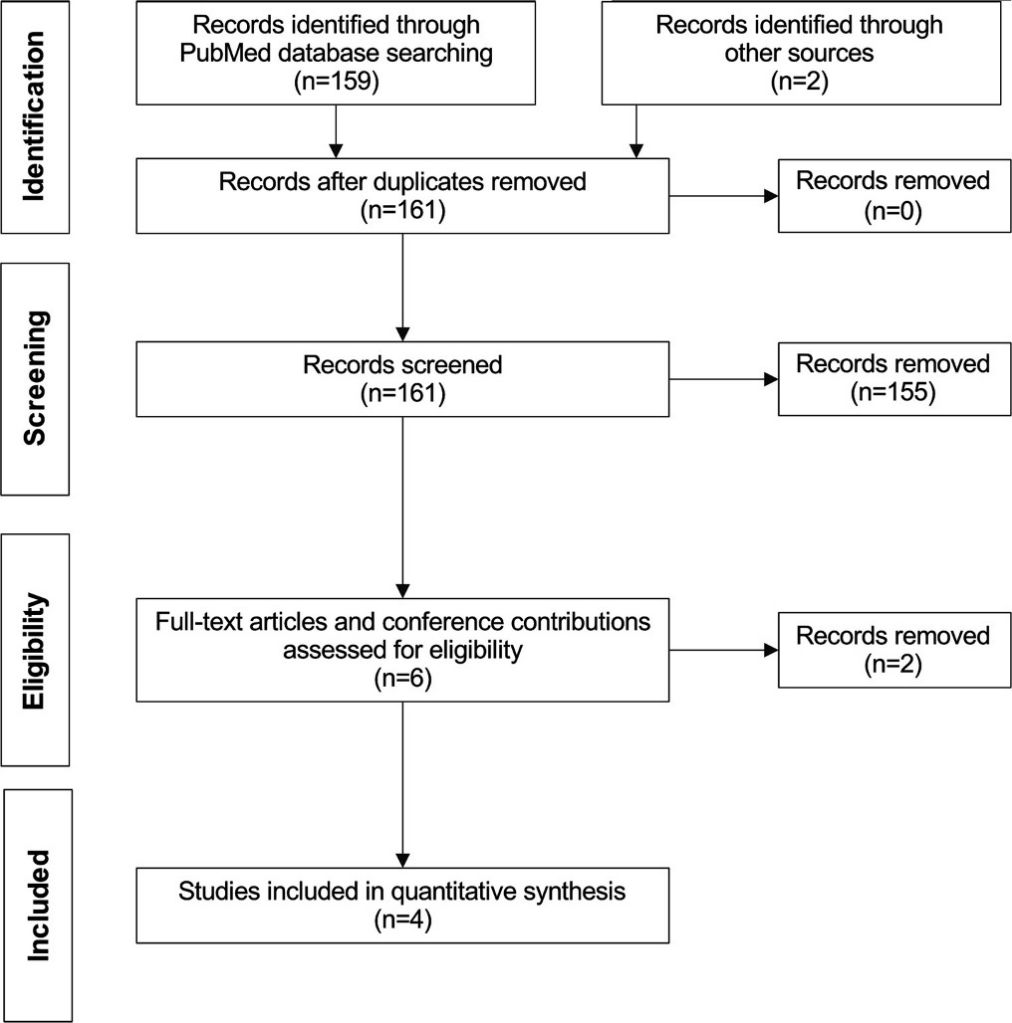
Flow diagram

This meta-analysis is reported according to the 2020 Preferred Reporting Items for Systematic Reviews and Meta-Analyses guidelines (PRISMA). A systematic literature search was conducted through the online database PubMed to identify RCTs which [1] assessed the treatment effect of EVT in acute ischemic stroke with established large infarct (defined by ASPECTS of ≤ 5 or perfusion imaging), and [2] reported the treatment effect for the primary outcome in patients with ASPECTS of 0–2 (Fig. 1). The keywords “stroke”, “Alberta Stroke Program Early Computed Tomography Score OR ASPECTS”, and “endovascular thrombectomy OR endovascular therapy” were used with “clinical trial” as the study design filter and a publication date from 1 January 2015. The last literature search was performed on 23 December 2023.

Data on the primary outcome were extracted by screening the eligible publications and their supplemental materials. Preliminary results from the TESLA trial were screened as reported in a preprint [6]. Preliminary results from the LASTE trial were extracted as presented at the annual meeting 2023 of the Society of Vascular and Interventional Neurology (SVIN) [5]. Ethical approval was not required for this meta-analysis.

### Statistical Analysis

The primary outcome was the shift toward better functional outcomes on the 90-day modified Rankin Scale (mRS). We conducted a generic inverse variance meta-analysis to assess the pooled odds ratio and its 95% confidence interval (CI) for the primary outcome. Statistical analyses were performed using R statistical software (version 4.3.0, R Project for Statistical Computing; package: *meta*).

## Results

We identified six RCTs which assigned patients with acute ischemic stroke and established large infarct to receive endovascular thrombectomy plus medical management or medical management alone. RESCUE-Japan LIMIT and TESLA were excluded for missing data on the subgroup of patients with ASPECTS 0–2 [1, 6]. Four trials met the inclusion criteria: SELECT2, ANGEL-ASPECT, TENSION, and LASTE [2–5]. Main characteristics of the eligible trials are shown in Table 1.

**Table 1:** Study designs of eligible randomized controlled trials

	SELECT2	ANGEL-ASPECT	TENSION	LASTE
*NCT number*	NCT03876457	NCT04551664	NCT03094715	NCT03811769
*Participating country (ies)*	USA, Canada, Europe, Australia, New Zealand	China	Europe, Canada	France, Spain
*Imaging modality*	CTP (98.3%)	NCCT	NCCT (82.2%)	MRI (83.6%)
	MRI (1.7%)	CTP	MRI (17.8%)	NCCT (16.4%)
*Definition of large core*	ASPECTS 3–5 or core volume ≥ 50 ml	ASPECTS 3–5 or core volume 70–100 ml	ASPECTS 3–5	< 80y: ASPECTS 0–5
				≥ 80y: ASPECTS 4–5
*Inclusion criteria*				
Time window	≤ 24h (to thrombectomy)	≤ 24h (to thrombectomy)	≤ 11h (to randomization)	≤ 6h 30 min or negative FLAIR
Age	18–85y	18–80y	≥ 18y	≥ 18y
Prestroke mRS	0–1	0–1	0–2	0–1
Admission NIHSS	0–42	6–30	0–26	0–42
*N total*	352	456	253	324
*N ASPECTS 0–2*	20	62	38	181
	/352 (5.6%)	/456 (13.6%)	/253 (15.0%)	/324 (55.9%)
*Primary outcome*	90-day mRS shift	90-day mRS shift	90-day mRS shift	90-day mRS shift

Abbreviations: *CTP* computed tomography perfusion; *MRI* magnetic resonance imaging; *NCCT* non-contrast computed tomography; *ASPECTS* Alberta Stroke Program Early CT Score; *FLAIR* fluid attenuated inversion recovery; *mRS* modified Rankin Scale; *NIHSS* National Institutes Health Stroke Scale

The eligible trials included a total of 1485 patients, of which 301 patients were enrolled with ASPECTS of 0–2. The percentage of patients with ASPECTS of 0–2 was 5.6% (20/352) in SELECT2, 13.6% (62/456) in ANGEL-ASPECT, 15.0% (38/253) in TENSION, and 55.9% (181/324) in LASTE. The trials did neither provide demographic, clinical, imaging nor treatment characteristics for the subgroup of patients with ASPECTS of 0–2.

**Fig. 2:**
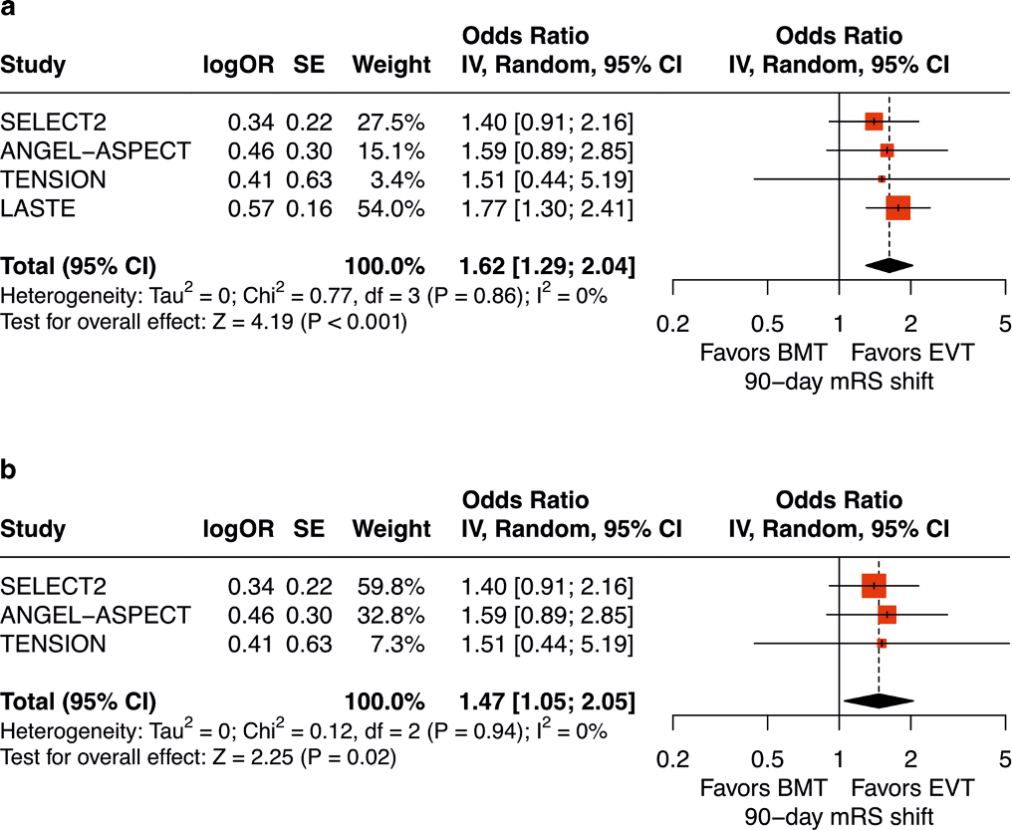
Random-effects meta-analysis to investigate the 90-day mRS shift in patients with ASPECTS of 0–2 who received endovascular thrombectomy compared with medical management alone. **a** Forest plot displaying the meta-analysis of four RCTs. The pooled results suggested a significant treatment effect of EVT for patients with ASPECTS of 0–2 (pooled odds ratio, 1.62, 95% CI, 1.29–2.04, *P* < 0.001). **b** Same as (**a**) after exclusion of LASTE. The pooled analysis of trials using CT as primary imaging modality also favored EVT (pooled odds ratio, 1.47, 95% CI, 1.05–2.05, *P* = 0.02)

The pooled analysis of the four RCTs showed a significant shift toward better 90-day mRS scores in favor of EVT compared with medical management alone in patients with ASPECTS of 0–2 (pooled odds ratio, 1.62, 95% CI, 1.29–2.04, *P* < 0.001) (Fig. 2a).

The LASTE trial contributed 60.1% of patients with ASPECTS of 0–2 for this meta-analysis. A sensitivity analysis of SELECT2, ANGEL-ASPECT, and TENSION, which enrolled patients using CT as primary imaging modality, also favored EVT in patients with ASPECTS of 0–2 (pooled odds ratio, 1.47, 95% CI, 1.05–2.05, *P* = 0.02) (Fig. 2b).

## Discussion

The present meta-analysis of four RCTs points to a treatment effect of EVT in specific patients with acute ischemic stroke and very low ASPECTS of 0–2. The sensitivity analysis of three RCTs with CT as primary imaging modality also favored EVT for established large infarcts with ASPECTS of 0–2. Together with the results from the HERMES collaboration, EVT might be efficacious across the full spectrum of ASPECTS ranging from 0–10 [1–5, 7]. This hypothesis challenges current treatment selection and imaging workflows in acute ischemic stroke due to large vessel occlusion.

The main finding is in line with the LASTE trial, which suggests a treatment effect of EVT in patients with ASPECTS of 0–2 [5]. Notably, a very low ASPECTS rating does not per se preclude the existence of salvageable brain volume. The benefit of EVT might be explained by the lowering of final infarct volume, which was found for patients with ASPECTS of 0–2 in a secondary analysis of the ANGEL-ASPECT trial. [8] In addition, mechanical reperfusion might have beneficial effects beyond penumbral salvage, such as the edema reduction per infarcted brain volume [9].

The potential treatment effect of mechanical reperfusion in ASPECTS 0–2 questions the withholding of EVT based on the ASPECTS rating in a large subgroup of stroke patients. If confirmed by individual patient data, this finding could have major clinical implications for the interhospital transfer and imaging workflows in patients with acute ischemic stroke and suspected large vessel occlusion. There might be less concerns about infarct progression as potential exclusion criterion, additionally supporting a low-threshold transfer from primary to thrombectomy-capable centers. In thrombectomy-capable centers, conventional CT/MRI for thorough identification of a large ischemic lesion might be no longer required in many stroke patients, and direct transfer to angiography suite could become standard practice. In addition, the role of perfusion imaging to select patients with low ASPECTS for EVT remains debatable. Recently, the TENSION trial provided evidence that in patients with ASPECTS of 3–5, EVT can be guided solely by non-contrast CT/MRI without perfusion imaging within 12 h after onset [4]. Subgroup analyses from SELECT2 are supportive of a persistent treatment effect in patients with very large ischemic core and in patients without target mismatch profile [2]. A benefit of EVT in ASPECTS of 0–2 would further question the added value of perfusion imaging for treatment selection in large ischemic strokes.

Nonetheless, the baseline ASPECTS might still be useful to anticipate the expected effect size of EVT for given stroke patients. The comparison of expected effect sizes between patients could gain more importance in the future, as the increasing number of patients eligible for EVT threatens to exceed the treatment capacity of many health care systems.

This study has certain limitations. Results from the LASTE trial were used as presented at SVIN 2023, and might differ in the final publication. Moreover, the large core trials must be interpreted in due consideration of the study inclusion and exclusion criteria. For instance, this meta-analysis was dominated by the LASTE trial, in which patients with ASPECTS of 0–2 were younger than 80 years, and were largely selected based on MRI in the early time window. Importantly, the sensitivity analysis of SELECT2, ANGEL-ASPECT, and TENSION also favored EVT. However, beyond this study-level meta-analysis, it will be of outmost importance to investigate the treatment effect of EVT in different subgroups of patients with ASPECTS of 0–2 in an individual patient data meta-analysis.

## Conclusion

EVT might be efficacious and superior to medical treatment alone in specific patients with ASPECTS of 0–2, challenging the use of ASPECTS for treatment selection in acute ischemic stroke due to large vessel occlusion.

## Data Availability

All data generated and analyzed in this study are provided in this report.
